# How Returning Aggregate Research Results Impacts Interest in Research Engagement and Planned Actions Relevant to Health Care Decision Making: Cohort Study

**DOI:** 10.2196/10647

**Published:** 2018-12-21

**Authors:** Elissa R Weitzman, Kara M Magane, Lauren E Wisk

**Affiliations:** 1 Division of Adolescent/Young Adult Medicine Boston Children's Hospital Boston, MA United States; 2 Computational Health Informatics Program Boston Children’s Hospital Boston, MA United States; 3 Department of Pediatrics Harvard Medical School Boston, MA United States

**Keywords:** aggregate research results, decision making, juvenile idiopathic arthritis, patient engagement, patient-reported outcome measures, rheumatic disease

## Abstract

**Background:**

Collection of patient-reported outcomes measures (PROs) may augment clinical data and inform health research, improving care, yet approaches to sustaining interest among patient cohorts in research participation are needed. One approach may involve returning aggregate research results (ARRs), which may help patients contextualize personal experiences, prompt conversations with providers or family, and encourage information seeking. This model has been demonstrated for Web-based patient-centered registries. Studies with clinical cohorts may further elucidate the model, its impacts on interest in research participation and planned actions, and potential for participants to experience this as helpful or harmful—gap areas.

**Objective:**

We sought to investigate the impacts of returning ARRs comprising summaries of PROs and clinical metrics to parents of children with rheumatic disease, assessing interest in future research participation among parents who viewed ARRs and plans for acting on returned information. Further, we sought to investigate reactions to viewing ARRs and how these reactions impacted planned actions.

**Methods:**

Clinical and PRO data were obtained about children in a national clinical disease registry, summarized, and processed into annotated infographics, comprising ARRs for children’s parents. Parents who viewed ARRs (n=111) were surveyed about the information’s perceived value and their reactions. Reaction patterns were summarized using principal components analysis (PCA), and associations among reaction patterns and interest in research participation and planned actions were estimated using multivariate logistic regression.

**Results:**

Parental endorsement of the value of ARRs for understanding their child’s condition and making care decisions was high (across 10 topics for which ARRs were shared, 42.2%-77.3% of the parents reported information was “very valuable”). Most (58/111, 52.3%) parents reported being more interested in participating in research after viewing ARRs, with the remainder reporting that their interest levels were unchanged. Reactions to viewing ARRs reflected experiencing validation/affirmation and information burden based on PCA. Reactions were not associated with child demographic or clinical characteristics and PROs, except that parents from households with less education reported greater information burden than those from more educated households (*P*=.007). In adjusted models, parents with higher validation/affirmation scores had increased odds of reporting heightened interest in research participation (adjusted odds ratio [AOR] 1.97, 95% CI 1.18-3.30), while higher information burden scores were associated with decreased odds of planned discussions with their child (AOR 0.59, 95% CI 0.36-0.95) and increased odds of planned discussions with providers (AOR 1.75, 95% CI 1.02-3.00).

**Conclusions:**

Returning ARRs may foster a “virtuous cycle” of research engagement, especially where ARRs are experienced favorably and affect plans to share and discuss ARRs in support of a child’s chronic disease care and treatment. Reactions to ARRs vary with education level, underscoring the need for attention to equity for this model.

## Introduction

Growing evidence supports the importance of engaging patients in research to share information about their disease and treatment experiences and health-related quality of life [1,2]. Where shared data flow into health care systems and clinical epidemiologic studies, better symptom management, improved treatments, and better outcomes result [3]. In support of engaging patients in sharing high-quality data, considerable investment has been made in developing standardized patient-reported outcomes measures (PROs) that characterize aspects of patients’ physical, mental, and social health [3-6]. PROs enable scientific rigor and help capture the patient voice, consistent with the paradigm shifting efforts to advance patient-centered outcomes research [7-10]. Yet, open questions remain about how to motivate ongoing patient engagement in research, and this question is central to ambitious efforts to activate vast cohorts in donating PROs and clinical data [11].

One possible approach is to operationalize a process whereby patients donate health data that are subsequently processed and returned in aggregate. Here, the return of aggregate research results (ARRs) about the cohort is hypothesized to motivate a virtuous cycle of data donation that can help grow the evidence base to advance more acceptable, effective therapies [12]. Viewing ARRs may be motivating for research participation if the returned information helps patients appraise personal experiences of disease and treatment [13] and informs conversations with health care providers and family members, factors that are relevant to health care decision making [14]. These actions, which reflect an engaged and activated patient and socially embedded nature of health care decision making, are central to models of chronic illness care [15-17]. These factors are also consistent with survey reports about what motivates sharing of personal health information [18-20] and are reflected in the appeal of Web-based patient-centered health information repositories [17]. Additional empirical testing of this “closed-loop” model is essential for ascertaining whether it fosters interest in research participation among clinical cohorts and to better understand its potential for being experienced as helpful or harmful—gap areas that are central to ensuring equipoise.

Returning ARRs may be informative and reassuring for some research participants, providing a normalizing context around experiences; ARRs may also be overwhelming and disquieting for others, including if ARRs show evidence of problems experienced by others with the same condition. Other factors, including the level of education and health literacy, might also affect acceptability. Concern about the balance of benefit or harm experienced when viewing ARRs may be especially acute for conditions that are rare, have treatments that rest on an immature evidence base or incompletely ameliorate symptoms or health-related quality of life, and pose risks for side effects [21]. Arguably, motivating ongoing research engagement for such conditions is especially important because gaps in knowledge might be filled, driving improvements in therapies at the system level and decision making at the patient or family level.

Pediatric-onset rheumatic disease (RD) is a trenchant case for examining these issues. Among youth, RD is rare but rising in incidence [22], with affected children facing significant hurdles regarding health-related quality of life due to the chronic relapsing nature of RD, unpredictable disease course, and difficult treatments [1-3]. Lack of a mature evidence base guiding RD care makes maintaining patient engagement in research especially important for gathering information about disease and treatment experiences to improve care and outcomes. For example, in a prior study focused on youth with RD, we found that treatment-related problems for standard RD therapies were common and contributed to poor health-related quality of life even after controlling for patient clinical characteristics and ameliorative effects of these treatments on symptoms, such as pain [23].

This study aims to investigate impacts of returning ARRs on interest in participating in future research within a larger project focused on investigating pediatric-onset RD. We engaged parents of children with RD in donating PROs about their child’s health and treatment experiences and then returned cohort-level summaries of clinical measures and PROs to parents, testing whether viewing these ARRs increased parents’ interest in future research participation and their intentions to discuss ARRs with others or seek further information. Such discussions might encourage shared decision making, consistent with theory [6,24]. We hypothesized that the receipt of ARRs would be highly motivating for future research engagement and that reactions to and planned actions arising from viewing ARRs would vary with participants’ experiences of benefit and burden.

## Methods

### Study Design and Setting

We obtained data from The Learning Cohort (TLC) study [23], which surveyed consented parents of youth with a pediatric-onset RD enrolled in the Childhood Arthritis and Rheumatology Research Alliance (CARRA) Registry [25] during subspecialty care visits. Surveys included PROs to capture aspects of disease or treatment experience and well-being [23]. PRO survey measures were programmed in the Research Electronic Data Capture system [25], from whence they could flow into the registry using a modular, ontology-based, federated informatics infrastructure constructed from open-source software; this infrastructure provides research investigators full ownership and access to their contributed data while supporting permissioned and robust data-sharing across federated sites [26]. In total, 4 CARRA Registry sites participated in this study, with Institutional Review Board approval from each. All participants provided informed consent. Details of the TLC study are published elsewhere [23].

**Figure 1:**
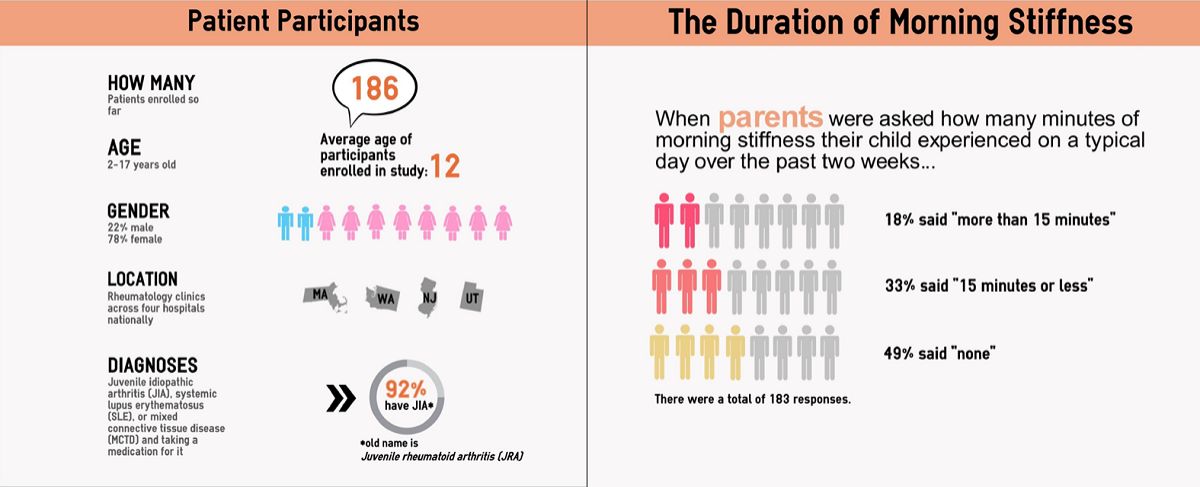
Example content from aggregate research results returned to parents.

Toward the close of the study period, the research team aggregated PRO data from the full TLC cohort (N=202 dyads) and clinical metrics from the CARRA Registry. These results were summarized by the research team into a curated, annotated set of ARRs, reflecting areas of leading concern to parents of registry-involved youth based on an initial survey of their information needs conducted when they enrolled [23]. ARRs comprised 68 unique slides (including titles and section headers) delivered on a tablet computer at a routine visit and were delivered as static infographics (visuals, figures, and charts). Topics covered study methods, clinical and treatment characteristics of children in the cohort, including medications used, patterns of health-related quality of life, experiences of pain and morning stiffness, and treatment problems (Figure 1). All materials were pretested, including the ARR slides, and the survey that was to be administered to parents to elicit reactions to returned data. The process was iterative to address all concerns. Pretests for accessibility were conducted with 5 parent volunteers and a representative of a family-based disease advocacy group. Pretests for accuracy and safety were conducted with 6 pediatric rheumatologists and 1 pediatric emergency room physician/ informatician.

From August 2015 to February 2016, *in lieu* of collecting additional PROs during clinic visits, an approximately 50% convenience sample of participating parents viewed ARRs (119 were approached, 115 consented; 96.6% consent rate), after which they completed a survey about their reactions to these materials; 111 parents provided complete data on their reactions to ARRs.

### Measures

#### Demographic, Clinical and Health Characteristics

Parents reported their child’s age; sex, race/ethnicity; diagnosis (juvenile idiopathic arthritis, systemic lupus erythematosus, or mixed connective tissue disease); overall health status; health-related quality of life [27]; pain interference [28]; morning stiffness; experience of serious side effects from a medication; methotrexate intolerance [29]; and highest education attained in the family. Disease duration was obtained from the CARRA Registry. Time in cohort was calculated as the number of days from the enrollment date (initial PRO collection date) to the final PRO collection date. Sample mode and mean imputation were used for 9 participants with incomplete data on demographic or clinical characteristics.

#### Perceived Value of and Reactions to the Return of Aggregate Research Results

Novel measures were developed and used to assess parents’ reactions to the return of ARRs. Perceived value of the return of ARRs was ascertained with the question, “Overall, how valuable might this summary information be when understanding and making decisions about your child’s condition and care?” asked for each of 10 topics that were shared in ARRs. Responses were given using a 3-point Likert scale (very valuable, somewhat valuable, and not valuable) and reported as frequencies. Sample size for each item ranged from 106 to 111 due to participant nonresponse on select items. Parents’ reactions to seeing ARRs were determined by the extent to which they agreed or disagreed with the following 6 statements, each rated on a 5-point Likert scale (strongly agree, agree, undecided, disagree, strongly disagree): “Seeing summary information about the experiences of other study participants was comforting because it made me feel like my experiences are shared and validated as real;” “Overall, seeing summary information about other patients’ experiences help me understand my child’s experience;” “Reviewing this type of information is within my comfort zone;” “Overall, this type of information raises more questions than it answers;” “This type of information requires more knowledge or expertise than I have to understand it;” and “I prefer to let my rheumatologist digest this type of information.” The mean of nonmissing items within the scale was used to impute missing response for 5 participants missing 1 or 2 (of 6) items.

#### Engagement Outcomes

Parents were asked to report their interest in participating in research studies after seeing ARRs; options included more interested, less interested, and not any more or less interested. As one respondent endorsed “less interested,” this response was combined with “not any more or less interested” to create a dichotomous variable. Parents were asked to report their planned actions after reviewing the summary information provided in ARRs by selecting all that applied from the following list: (1) discuss contents of the slide-deck with my child’s health care provider; (2) discuss contents of the slide-deck with my child; (3) explore different medications; (4) look up information about something I saw in this slide-deck; and (5) do something else.

### Statistical Analyses

All analyses were conducted using SAS 9.4 software (SAS Institute, Inc, Cary, North Carolina). Summary statistics were generated to describe sample characteristics; differences in demographic and clinical characteristics between parents who did (n=111) or did not (n=91) receive ARRs were evaluated using *t* tests, Kruskal-Wallis, or chi-square (χ²) tests, as appropriate. Principal components analysis (PCA) was conducted to investigate the commonalities between ARR reactions and generate summary variables based on individual reaction measures. All factors with eigenvalues >1 were retained consistent with the standard practice [30,31], leading to a 2-factor solution, and an orthogonal rotation was applied to generate 2 uncorrelated scales (hereafter referred to as “validation/affirmation” and “information burden”).

Validation/affirmation reflects reactions to ARRs that reflect experiences of recognizing the legitimacy of personal experiences, greater insight into their child’s condition, and level of comfort with ARRs. Information burden reflects reactions to ARRs that reflect experiences akin to being over one’s head or uncomfortable with information in ARRs owing to perceived lack of personal expertise, preferences for their provider to digest the information and uncertainty. Pearson correlations, *t* tests, and analysis of variance (as appropriate) were used to examine bivariate relationships between each of the 2 ARR reaction constructs and demographic and clinical characteristics. Subsequently, multivariate logistic regression was used to model engagement outcomes as predicted by the 2 ARR reaction constructs; both unadjusted (controlling only for both factors simultaneously) as well as models adjusting for child age, race/ethnicity, and parent education were used.

## Results

### Sample Characteristics

Children in this cohort were 12.0 years of age on average (SD 3.6) and predominantly female (n=161, 79.7%), diagnosed with juvenile idiopathic arthritis (n=187, 92.6%), white/non-Hispanic individuals (n=152, 75.2%), and had parents with any college education (n=144, 71.3%); the average disease duration was 7.7 (SD 3.5) years. No differences were observed in demographic or clinical characteristics between those who did or did not receive ARRs (Table 1).

### Perceived Value of and Reactions to the Return of Aggregate Research Results

**Figure 2:**
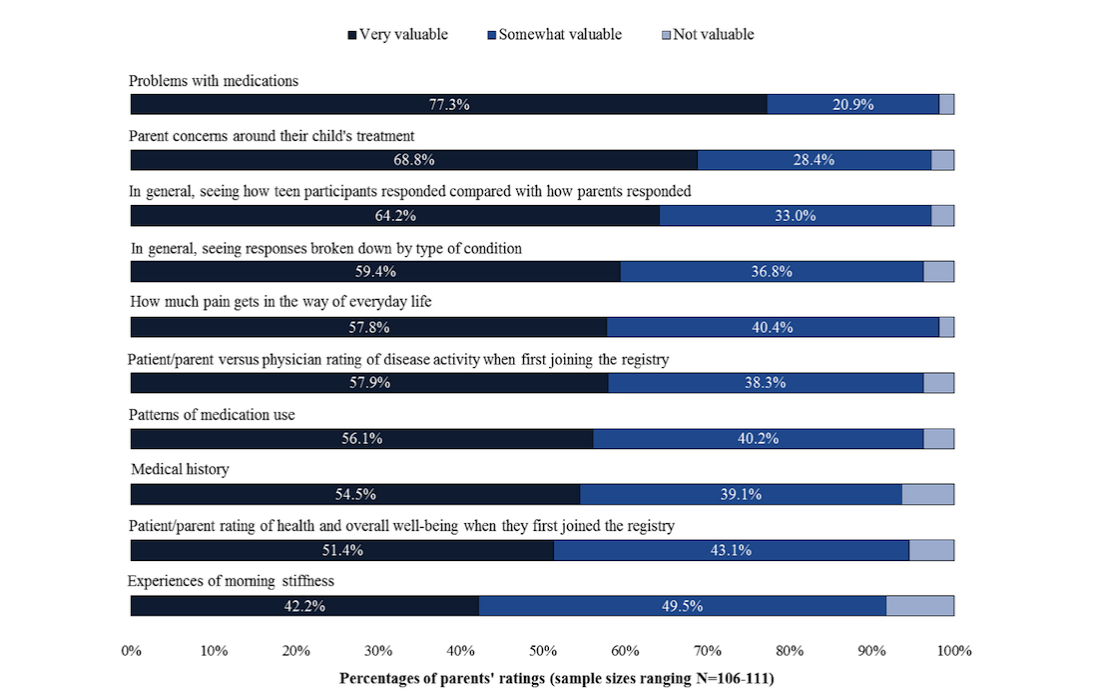
Parents’ ratings of the value of each patient-reported topic presented in aggregate research results for understanding and making decisions regarding their child’s condition and care.

The proportion of parents finding the ARR topics to be “very valuable” ranged from 42.2% (for experiences of morning stiffness) to 77.3% (for medication problems; Figure 2).

**Figure 3:**
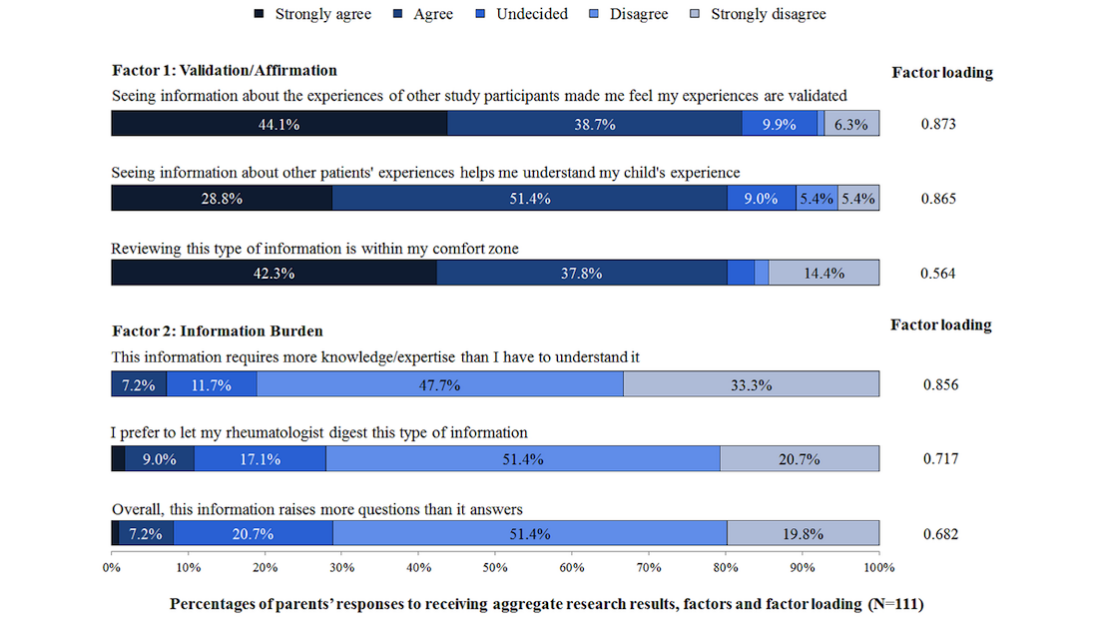
Percentages and factor loadings of response items regarding reactions to the return of aggregate research results.

PCA identified a 2-factor solution with high loadings for all 6 ARR reaction items (Figure 3).

The validation/affirmation construct was defined by 3 items reflecting having one’s experience validated (factor loading=0.873), improved understanding of their child’s condition (factor loading=0.865), and feeling that the ARR materials are within their comfort zone (factor loading=0.564). The information burden construct was defined by reports of requiring more knowledge to understand ARRs (factor loading=0.856), preferring a physician to “digest” ARRs (factor loading=0.717), and viewing ARRs raising more questions than were answered (factor loading=0.682). Parents from households with less education reported greater information burden than those from more educated households (*P*=.007; Table 2); factors were not significantly associated with other demographic or clinical characteristics.

### Engagement Outcomes

The majority of the parents (58/111, 52.3%) reported that after seeing ARR, they were more interested in participating in research. Higher validation/affirmation scores were associated with nearly a 2-fold increase in the odds of reporting more interest in research participation (adjusted odds ratio [AOR]: 1.97, 95% CI 1.18-3.30; Table 3). Nearly one-third (35/111, 31.5%) of the parents reported that they would discuss the contents of ARRs with their child, nearly one-fifth (20/111, 18.0%) reported they would look up information, 15.3% (17/111) said that they would discuss ARR contents with their child’s health care provider, and approximately one-tenth (11/111, 9.9%) reported they would explore different medications. Parents who reported greater information burden were less likely to report wanting to discuss the contents of ARRs with their child (AOR 0.59, 95% CI 0.36-0.95) but were more likely to report wanting to discuss the contents of ARRs with their child’s health care provider (AOR 1.75, 95%CI 1.02-3.00).

**Table 1:** The parent-reported sample characteristics by receipt of aggregate research results (ARRs).

Characteristic			Total (N=202)	Received ARRs (n=111)	Did not receive ARRs (n=91)	*P* value
**Demographics**						
	Child age (years), mean (SD)		12.0 (3.6)	11.7 (3.6)	12.3 (3.7)	.29
	**Child sex, n (%)**					.87
		Female	161 (79.7)	88 (79.3)	73 (80.2)	—^a^
		Male	41 (20.3)	23 (20.7)	18 (19.8)	—
	**Child race or ethnicity, n (%)**					.63
		White, non-Hispanic	152 (75.2)	85 (76.6)	67 (73.6)	—
		Non-white or Hispanic	50 (24.8)	26 (23.4)	24 (26.4)	—
	**Highest level of parent education, n (%)**					.79
		High school graduate or less	58 (28.7)	31 (27.9)	27 (29.7)	—
		Any college	144 (71.3)	80 (72.1)	64 (70.3)	—
**Clinical characteristics**						
	**Diagnosis, n (%)**					.50
		Juvenile idiopathic arthritis	187 (92.6)	104 (93.7)	83 (91.2)	—
		Systematic lupus erythematosus or mixed connective tissues disease	15 (7.4)	7 (6.3)	8 (8.8)	—
	Disease duration (in years), mean (SD)		7.7 (3.5)	7.7 (3.5)	7.7 (3.6)	.99
	**Methotrexate use and intolerance, n (%)**					.65
		No methotrexate use	103 (51.0)	59 (53.2)	44 (48.4)	—
		Use with no intolerance	60 (29.7)	30 (27.0)	30 (33.0)	—
		Methotrexate intolerance	39 (19.3)	22 (19.8)	17 (18.7)	—
	Overall health^b^, mean (SD)		8.1 (2.0)	8.2 (1.8)	8.0 (2.3)	.45
	**Typical morning stiffness in the past 2 weeks (minutes)^c^**					.30
		>15	36 (17.8)	17 (15.3)	19 (20.9)	—
		≤15	166 (82.2)	94 (84.7)	72 (79.1)	—
	**Lifetime serious medication side effects**					.64
		One or more	52 (25.7)	30 (27.0)	22 (24.2)	—
		None	150 (74.3)	81 (73.0)	69 (75.8)	—
	**Pediatric Quality of Life Inventory 4.0 scores, mean (SD)**					
		Total score	75.7 (18.3)	74.9 (18.4)	76.7 (18.4)	.50
		Psychosocial score	76.1 (18.0)	75.2 (18.2)	77.1 (17.8)	.46
		Physical score	75.1 (22.6)	74.4 (22.3)	75.9 (23.1)	.65
	Patient-Reported Outcomes Measurement Information System Pain Interference^e^, mean (SD)		50.5 (10.9)	51.2 (11.0)	49.7 (10.9)	.35
	Time in cohort (days), mean (SD)		95.8 (100.9)	105.1 (100.7)	84.3 (100.4)	.14

^a^Not applicable.

^b^Parents’ rating of their child’s overall health from 1 to 10, where higher scores indicate better health.

^c^Parents’ report of the number of minutes of morning stiffness their child experiences on a typical day over the past 2 weeks.

^d^The possible range of scores is from 0 to 100, with higher score indicating better quality of life.

^e^Raw pain interference scores were transformed into a “*t* score” for each participant. The *t* score rescales the raw score into a standardized score with a mean of 50, SD of 10, and the possible range of 38-78, with higher score indicating more pain interference.

**Table 2:** Reactions to aggregate research results (ARRs) by demographic and clinical characteristics (n=111).

Characteristic			Factor 1: Validation/affirmation^a^		*P* value		Factor 2: Information burden^b^		*P* value	
**Demographic characteristics**										
	Child age (years), correlation coefficient			0.12		.21		0.00		.98
	**Child sex, mean (SD)**									
		Female		–0.05 (1.02)		.29		0.07 (1.02)		.15
		Male		0.20 (0.92)		—^c^	–0.27 (0.89)		—
	**Child race or ethnicity, mean (SD)**									
		White, non-Hispanic		–0.01 (1.05)		.92		–0.06 (0.96)		.24
		Non-white or Hispanic		0.02 (0.81)		—	0.20 (1.11)		—
	**Highest level of parent education, mean (SD)**									
		High school graduate or less		0.07 (0.89)		.67		0.41 (1.10)		.007
		Any college		–0.03 (1.04)		—	–0.16 (0.92)		—
**Clinical characteristics**										
	**Diagnosis, mean (SD)**									
		Juvenile idiopathic arthritis		–0.02 (1.02)		.52		0.01 (0.99)		.69
		Systematic lupus erythematosus or mixed connective tissues disease		0.24 (0.70)		—	–0.15 (1.15)		—
	Disease duration (years), correlation coefficient			0.11		.27		0.05		.57
	**Methotrexate use and intolerance, mean (SD)**									
		No methotrexate use		–0.01 (0.96)		.27		0.07 (0.97)		.54
		Use with no intolerance		0.20 (0.82)		—	–0.17 (1.01)		—
		Methotrexate intolerance		–0.25 (1.28)		—	0.04 (1.08)		—
	Overall health^d^, correlation coefficient			−0.07		.48		−0.03		.73
	**Typical morning stiffness in the past 2 weeks (minutes)^e^, mean (SD)**									
		>15		0.11 (1.05)		.61		0.05 (1.02)		.83
		≤15		–0.02 (1.00)		—	–0.01 (1.00)		—
	**Lifetime serious medication side effects, mean (SD)**									
		One or more		0.27 (0.95)		.09		–0.25 (0.78)		.10
		None		–0.10 (1.01)		—	0.09 (1.06)		—
	**Pediatric Quality of Life Inventory 4.0** **scores^f^, correlation coefficient**									
		Total		–0.09		.34		–0.11		.24
		Psychosocial Score		–0.11		.25		–0.10		.29
		Physical Score		–0.05		.61		–0.11		.26
	Patient-Reported Outcomes Measurement Information System Pain Interference^g^, correlation coefficient			0.06		.54		–0.02		.82
	Time in Cohort (days), correlation coefficient			–0.02		.80		0.08		.43

^a^Reflects the extent to which parents feel their experience is validated or affirmed when viewing ARRs; higher scores indicate greater agreement.

^b^Reflects the extent to which parents experienced information burden when viewing; higher scores indicate greater agreement.

^c^Not applicable.

^d^Parent’s rating of their child’s overall health from 1 to 10, where higher scores indicate better health.

^e^Parent’s report of the number of minutes of morning stiffness their child experiences on a typical day over the past 2 weeks.

^f^The possible range of scores is from 0 to 100, with higher score indicating better quality of life.

^g^Raw pain interference scores were transformed into a “*t* score” for each participant. The *t* score rescales the raw score into a standardized score with a mean of 50, SD of 10, and the possible range of 38-78. A higher score indicates more pain interference.

**Table 3:** Associations between reactions to aggregate research results and engagement outcomes.

Outcome^a^			Outcome prevalence, n (%)	Unadjusted models, OR (95% CI)	Adjusted models, OR (95% CI)
**More interest in participating in research**					
	Validation/affirmation		58 (52.3)	1.97 (1.21-3.18)	1.97 (1.18-3.30)
	Information burden			1.33 (0.89-1.98)	1.36 (0.89-2.09)
**Planned actions**					
	**Discuss with child**				
		Validation/affirmation	35 (31.5)	1.22 (0.79-1.87)	1.18 (0.75-1.86)
		Information burden		0.69 (0.45-1.07)	0.59 (0.36-0.95)
	**Look up information**				
		Validation/affirmation	20 (18.0)	0.93 (0.58-1.47)	0.87 (0.54-1.41)
		Information burden		0.68 (0.39-1.17)	0.64 (0.36-1.15)
	**Discuss with providers**				
		Validation/affirmation	17 (15.3)	0.69 (0.43-1.11)	0.69 (0.42-1.13)
		Information burden		1.63 (0.98-2.71)	1.75 (1.02-3.00)
	**Explore different medications**				
		Validation/affirmation	11 (9.9)	1.03 (0.54-1.98)	1.06 (0.53-2.10)
		Information burden		1.15 (0.63-2.11)	1.31 (0.69-2.52)

^a^Frequency and unadjusted prevalence of engagement outcomes among those who received aggregate research results (n=111). Planned action prevalence did not sum to 100% as participants could endorse multiple actions. “Unadjusted” models controlled for validation/affirmation and information burden scales only. Adjusted models controlled for the child’s age, race/ethnicity, and highest education attained in the family in addition to both validation/affirmation and information burden scales.

## Discussion

This study provides evidence that returning ARRs to study participants who have donated data is highly motivating for ongoing research participation. By investigating effects of sharing ARRs on interest in research participation and planned actions stemming from this experience, findings extend the understanding of benefits of patient engagement in health care research [32]. More than half of the parents in the cohort exposed to ARRs reported increased interest in research participation as a function of receiving ARRs—good news for participatory research models, including those predicated on engaging cohorts in donating data. Moreover, parents endorsed plans for proactive engagement in their child’s treatment and outcomes after receiving ARRs, with specific plans varying as a function of validation/affirmation and information burden. Overall, reactions to ARRs aligned with hopes for fostering motivation for research participation and activation in the health care process [33,34].

As hypothesized, the experience of viewing ARRs was not “one size fits all”—participants’ responses to the model reflected both experiences of validation/affirmation, wherein parents gained value from contextualizing disease or treatment experiences through viewing cohort-level data, and information burden, wherein aggregate results may be cognitively overwhelming. Yet, as greater information burden was associated with plans to discuss results with a provider, even parent participants who may have felt overwhelmed by ARRs might benefit if they are stimulated to talk with health care providers to understand findings and discuss any implications for their child. Such activities could foster improved patient-provider partnership and shared decision making. Notably, experiencing greater information burden was evident among participants who reported lower levels of educational attainment. As such, achieving goals of optimizing and equalizing health benefits and reducing the potential for disparities stemming from this model may require additional support around interpreting and processing ARRs [34]. Nationally, low levels of health literacy and numeracy indicate that large segments of the US population may face barriers to understanding health data, underscoring the importance of attending to these issues [35,36].

This investigation was undertaken with a cohort situated in a well-defined national multisite disease registry, whose members’ diagnoses were clinically confirmed—significant strengths. Still, several limitations merit mention. First, findings reflect the experiences of engaged parents of registry participants who viewed a specific set of ARRs and may not generalize to other populations that may differ in conditions, concerns, and experiences (including the history of benefit or harm from research and care); the ARR contents are also specific to the population. Second, this study leveraged the visit structure of a registry-engaged clinical cohort, layering data collection and return onto the natural visit cycle of this cohort. Additional research using a randomized trial design would inform comparisons of engagement outcomes for participants who did and did not receive ARRs over time. Third, participants shared structured PROs, some validated, others novel; items describing motivation to participate in research and experiences of receiving ARRs are not validated but capture important patient-centered dimensions of research experience. Fourth, parent proxy reports of child clinical characteristics and PROs and parent reactions to the return of ARRs may differ from those of the child [9,37], and results from this study should not be construed as reflecting child (ie, patient) reactions to the return of ARRs or reactions of another parent or guardian. Relatedly, excepting the measure of the highest parent education attained, demographic characteristics describe the child not parents. Congruence between child and parent experiences of this model and further investigation of effects of parent demographics on outcomes may further inform this work and merit future study. Lastly, self-reported data are subject to recall and social desirability biases; however, the use of structured and validated measures and electronic data collection help protect against known validity threats.

In sum, viewing ARRs increased motivation for research participation among a majority of study participants and shows promise for driving greater patient activation and engagement. Results of this study are encouraging in light of national plans for engaging volunteers in donating personally generated data, including PROs, to drive precision medicine and comparative effectiveness research [7,11,38,39]. To the extent that these efforts utilize a closed loop approach in which ARRs are returned to a learning and sharing cohort, they may thrive. Protecting against the potential for unintentionally worsening health disparities is vital as results show that participants from households with less parent education were more likely to experience information burden from viewing ARRs. Should this lead to the differential engagement or attrition of less educated participants, biases in study findings could be introduced and the ultimate fairness and beneficence of the model undermined.

## Abbreviations

AOR: adjusted odds ratio
ARR: aggregate research result
CARRA: Childhood Arthritis and Rheumatology Research Alliance
PCA: principal components analysis
PRO: patient-reported outcome measure
RD: rheumatic disease
TLC: The Learning Cohort
